# Supplementary material for: Empirical methods for controlling false positives and estimating confidence in ChIP-Seq peaks
Source: BMC Bioinformatics. 2008 Dec 5;9:523. doi: 10.1186/1471-2105-9-523 (PMC2628906; doi:10.1186/1471-2105-9-523)
Supplement: Additional File 1 — A variety of html documents from the USeq web site detailing the available applications, their best usage, output file type descriptions, command line menus, etc. [file 1471-2105-9-523-S1.zip › USeqUserGuides/JavaDoc.zip!/JavaDoc/bioroot/strain/package-tree.html]

bioroot.strain Class Hierarchy


|  |  |  |
| --- | --- | --- |
| **Overview** \| **Package** \| Class \| Use \| **Tree** \| **Deprecated** \| **Index** \| **Help** | |  |
| **PREV**   **NEXT** | **FRAMES**    **NO FRAMES**     **All Classes** |  |


---


## Hierarchy For Package bioroot.strain

**Package Hierarchies:**: All Packages

---

## Class Hierarchy

- java.lang.Object
  - javax.servlet.GenericServlet (implements java.io.Serializable, javax.servlet.Servlet, javax.servlet.ServletConfig)
    - javax.servlet.http.HttpServlet (implements java.io.Serializable)
      - bioroot.strain.**NewStrain**- bioroot.strain.**StrainBase**- bioroot.strain.**StrainModify**- bioroot.strain.**StrainSpreadSheet**- bioroot.strain.**StrainUpload**- bioroot.strain.**StrainViewPreferences**
    - bioroot.**Preference**
      - bioroot.strain.**StrainPreference**
      - bioroot.**ReagentBean** (implements java.lang.Comparable<T>)
        - bioroot.strain.**StrainBean**
        - bioroot.strain.**StrainUploadBean**

---


|  |  |  |
| --- | --- | --- |
| **Overview** \| **Package** \| Class \| Use \| **Tree** \| **Deprecated** \| **Index** \| **Help** | |  |
| **PREV**   **NEXT** | **FRAMES**    **NO FRAMES**     **All Classes** |  |


---
